# Digital quantum simulation of fermionic models with a superconducting circuit

**DOI:** 10.1038/ncomms8654

**Published:** 2015-07-08

**Authors:** R. Barends, L. Lamata, J. Kelly, L. García-Álvarez, A. G. Fowler, A Megrant, E Jeffrey, T. C. White, D. Sank, J. Y. Mutus, B. Campbell, Yu Chen, Z. Chen, B. Chiaro, A. Dunsworth, I.-C. Hoi, C. Neill, P. J. J. O'Malley, C. Quintana, P. Roushan, A. Vainsencher, J. Wenner, E. Solano, John M. Martinis

**Affiliations:** 1 Google Inc., Santa Barbara, California 93117, USA.; 2 Department of Physical Chemistry, University of the Basque Country UPV/EHU, Apartado 644, Bilbao E-48080, Spain.; 3 Department of Physics, University of California, Santa Barbara, California 93106, USA.; 4 Department of Materials, University of California, Santa Barbara, California 93106, USA.; 5 IKERBASQUE, Basque Foundation for Science, Maria Diaz de Haro 3, Bilbao 48013, Spain.

## Abstract

One of the key applications of quantum information is simulating nature. Fermions are ubiquitous in nature, appearing in condensed matter systems, chemistry and high energy physics. However, universally simulating their interactions is arguably one of the largest challenges, because of the difficulties arising from anticommutativity. Here we use digital methods to construct the required arbitrary interactions, and perform quantum simulation of up to four fermionic modes with a superconducting quantum circuit. We employ in excess of 300 quantum logic gates, and reach fidelities that are consistent with a simple model of uncorrelated errors. The presented approach is in principle scalable to a larger number of modes, and arbitrary spatial dimensions.

## Abstract (web summary)

Quantum simulation offers an unparalleled computational resource, but realizing it for fermionic systems is challenging due to their particle statistics. Here the authors report on the time evolutions of fermionic interactions implemented with digital techniques on a nine-qubit superconducting circuit.

## Graphical abstract

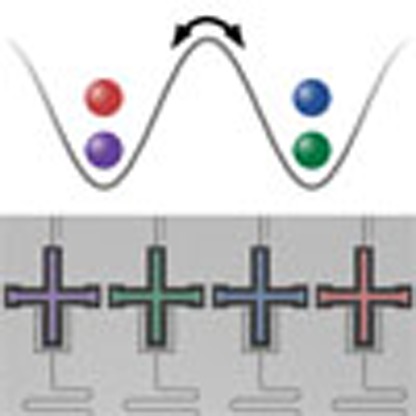

Simulating quantum physics with a device which itself is quantum mechanical, a notion Richard Feynman originated^1^, would be an unparallelled computational resource. However, the universal quantum simulation of fermionic systems is daunting due to their particle statistics^2^, and Feynman left as an open question whether it could be done, because of the need for physically implementing non-local control. Quantum simulation of fermionic models is highly desirable, as computing the properties of interacting particles is classically difficult. Determining static properties with quantum Monte Carlo techniques is already complicated due to the sign problem^3^, arising from anticommutation, and dynamic behaviour is even harder.

The key to quantum simulation is mapping a model Hamiltonian onto a physical system. When the physical system natively mimics the model, the mapping can be direct and simulations can be performed using analogue techniques. Already, fermionic models have been simulated at scale using large clouds of natively fermionic gases^4^,^5^. A complementary approach is digital quantum simulation^6^. It allows for constructing arbitrary interactions, and holds the promise that it can be implemented on an error-corrected quantum computer, but at the cost of many gates. However, the digital approach is in its infancy—so far, the only experiment is the simulation of a spin Hamiltonian in ion traps^7^—because it requires complex sequences of logic gates, especially for non-local control, which hinge on carefully constructed interactions between subsets of qubits in a larger system; a demanding task for any platform. A digital fermionic simulation can therefore be regarded as a hard test.

Here, we explore fermionic interactions with digital techniques^6^ in a superconducting circuit. Focusing on the Hubbard model^8^,^9^, we perform time evolutions with constant interactions as well as a dynamic phase transition with up to four fermionic modes encoded in four qubits, using the Jordan–Wigner transformation^10^. The implemented digital approach is universal and allows for the efficient simulation of fermions. The required number of gates scales only polynomially with the number of modes^9^, even with physical nearest-neighbour qubit coupling only. Moreover, the model system is not limited to the dimensionality of the physical system, allowing for the simulation of fermionic models in two and three spatial dimensions^9^,^11^. We use in excess of 300 single-qubit and two-qubit gates, to implement fermionic models that require fully, yet separately tunable 
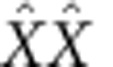
, 
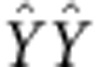
 and 
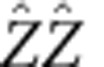
 interactions. We reach global fidelities that are limited by gate errors in an intuitive error model. These results are made possible by recent advances in architecture and control of superconducting qubits^12^,^13^,^14^. Our experiment is a critical step on the path to creating an analogue-digital quantum simulator—we foresee one using discrete fermionic modes combined with discrete^15^ or continuous^16^ bosonic modes, highlights the digital approach and is a demonstration of digital quantum simulation in the solid state.

## Results

### Implementing the Hubbard model with gates

**Figure 1:**
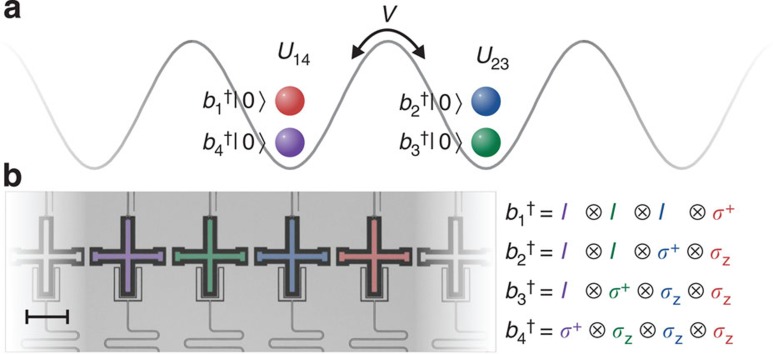
Model and device. (**a**) Hubbard model picture with two sites and four modes, with hopping strength *V* and on-site interactions *U*. The creation of one excitation from the groundstate is shown for each mode. (**b**) Optical micrograph of the device. The scale bar (bottom left) denotes 200 μm. The coloured cross-shaped structures are the used Xmon transmon qubits. The construction of the fermionic operators for four modes is shown on the right. Colours highlight the corresponding sites, qubits and operators.

At low temperatures, classes of fermionic systems can be accurately described by the Hubbard model. Here hopping (strength *V*) and repulsion (strength *U*) compete (see Fig. 1a), capturing the rich physics of many-body interactions such as insulating and conducting phases in metals^17^,^18^. The generic Hubbard Hamiltonian is given by: 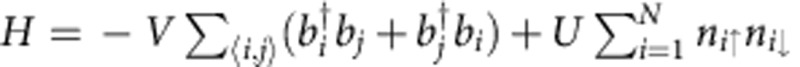, with *b* the fermionic annihilation operator and *i*,*j* running over all adjacent lattice sites. The first term describes the hopping between sites and the last term the on-site repulsion. It is insightful to look at a fermionic two-mode example,


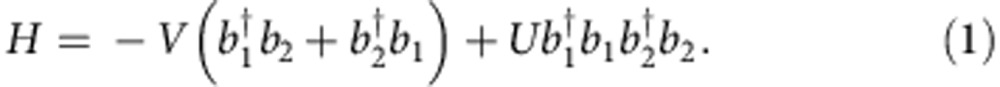


We can express the fermionic operators in terms of Pauli and ladder operators using the Jordan–Wigner transformation^10^: 
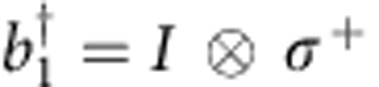
 and 
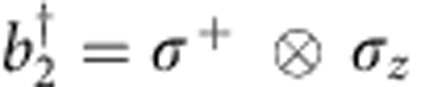
, where the *σ*_*z*_ term ensures anticommutation. In essence, we use non-local control and map a local fermionic Hamiltonian to a local spin Hamiltonian. The qubits act as spins, and carry the fermionic modes (Fig. 1a,b). A fermionic mode is either occupied or unoccupied, and spinless—the spin degree of freedom is implemented here by using four modes to simulate two sites with two spins. We note that for higher spatial dimensions this approach is still viable, the only difference is that the local fermionic Hamiltonian now maps to a non-local spin Hamiltonian, which can be efficiently implemented as recently shown^9^,^11^. Using the above transformation, the Hamiltonian becomes


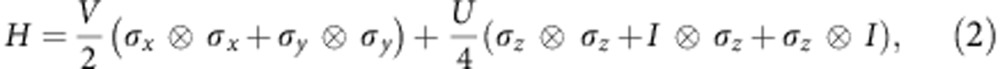


which can be implemented with separately tunable 
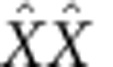
, 
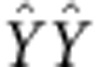
 and 
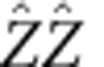
 interactions. Here we use the convention to map an excited fermionic mode |1〉 (excited logical qubit) onto a qubit's physical groundstate |*g*〉, and a vacuum fermionic mode |0〉 (ground logical qubit) onto a qubit's physical excited state |*e*〉.

Our experiments use a superconducting nine-qubit multipurpose processor, see Fig. 1b. Device details can be found in ref. 19. The qubits are the cross-shaped structures^20^ patterned out of an aluminium film on a sapphire substrate. They are arranged in a linear chain with nearest-neighbour coupling. Qubits have individual control, using microwave and frequency-detuning pulses (top), and readout is done through dispersive measurement (bottom)^21^. By frequency tuning of the qubits, interactions between adjacent pairs can be separately turned on and off. This system allows for implementing non-local gates, as it has a high level of controllability, and is capable of performing high-fidelity gates^12^,^22^. Importantly, single- and two-qubit gate fidelities are maintained when scaling the system to larger numbers of qubits, as shown by the consistency of errors with the five-qubit device^12^.

**Figure 2:**
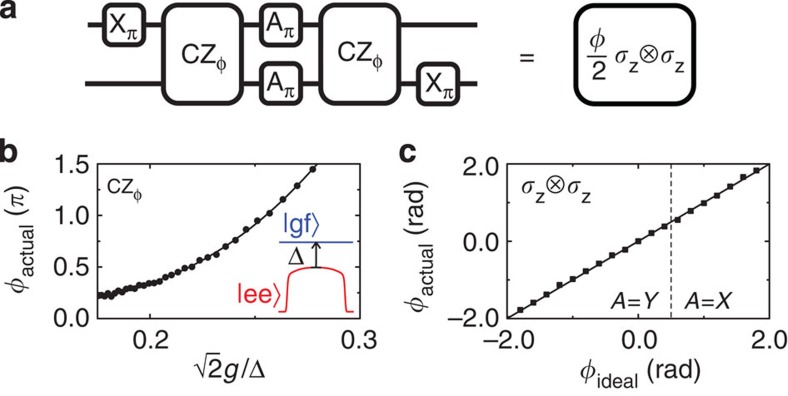
Gate construction. (**a**) Construction of the gate 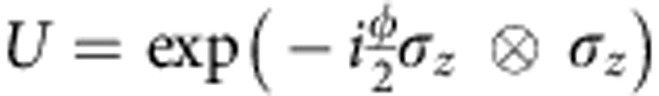 from single-qubit rotations and the tunable CZ_*φ*_-entangling gate. To enable small and negative angles, we include *π* pulses around the *x* axis (A=X) or *y* axis (A=Y). The unitary diagonals are (1 *e*^*iφ*^ *e*^*iφ*^ 1). (**b**) Tunable CZ_*φ*_ gate, implemented by moving |*ee*〉 (red) close to |*gf*〉 (blue). Coupling strength is *g*/2*π*=14 MHz, pulse length is 55 ns, and typically Δ/2*π*=0.7 GHz when idling. (**c**) Measured versus desired phase of the full sequence, determined using quantum state tomography.

The basic element used to generate all the interactions is a simple generalization of the controlled-phase (CZ) entangling gate (Fig. 2a,b). We implement a state-dependent frequency pull by holding one qubit steady in frequency and bringing a second qubit close to the avoided level crossing of |*ee*〉 and |*gf*〉 using an adiabatic trajectory^23^. By tuning this trajectory, we can implement a tunable CZ_*φ*_ gate. During this operation, adjacent qubits are detuned away in frequency to minimize parasitic interactions. The practical range for *φ* is 0.5–4.0 rads; below this range, parasitic 
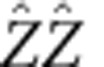
 interactions with other qubits become relevant, and above this range population starts to leak into higher-energy levels (see Supplementary Note 5 and refs 12, 19). Using single-qubit gates and two entangling gates, we can implement the tunable 
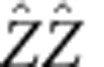
 interactions, as shown in Fig. 2c. In this gate construction, the *π*-pulses naturally suppress dephasing^24^.

### Verifying operator anticommutativity

**Figure 3:**
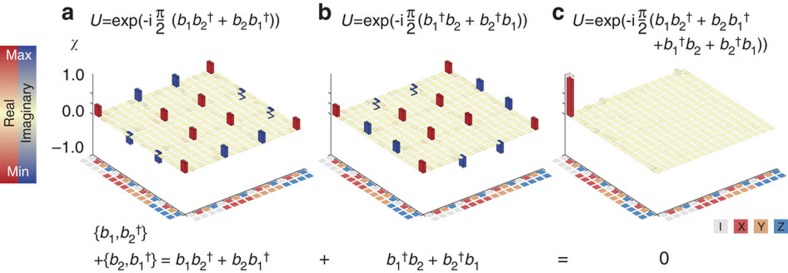
Quantum process tomography of operator anticommutation. The process matrices are shown for the non-trivial Hermitian terms of the anticommutation relations. (**a**) Process matrix of the unitary 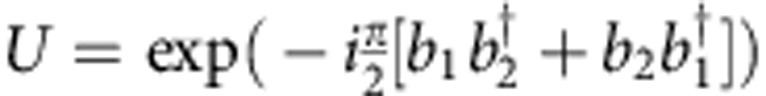. (**b**) Process matrix of the unitary 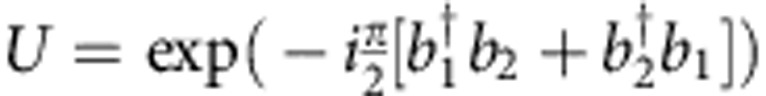. (**c**) The sequence of both processes, 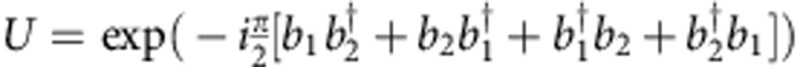, yields the identity. The significant matrix elements, red for the real and blue for the imaginary elements, are close to the ideal (transparent).

First, we have experimentally verified that the encoded fermionic operators anticommute, see Fig. 3, by implementing the following anticommutation relation 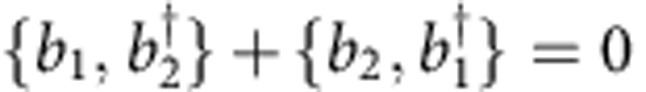. The latter can be separated into two non-trivial Hermitian terms: 
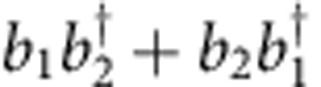
 (Fig. 3a) and 
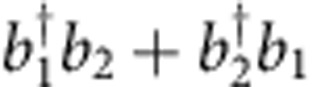
 (Fig. 3b). Their associated unitary evolution, 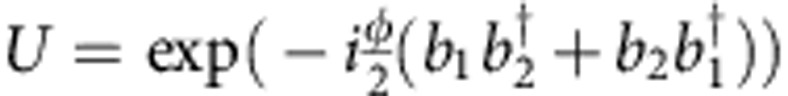 for the first one, has been implemented using gates with strength *φ*=*π*. The measured process matrices (*χ*) for these terms are determined using quantum process tomography, and constrained to be physical (Supplementary Note 2). We find that the processes are close to the ideal, with fidelities Tr(*χ*_ideal_*χ*)=0.95, 0.96. As the Hermitian terms sum up to zero, their unitary evolutions combine to the identity (Fig. 3c). We find that the sequence of both processes yields in fact the identity, as expected for anticommutation, with a fidelity of 0.91.

### Simulations with two fermionic modes

We now discuss the simulation of fermionic models. We use the Trotter approximation^25^ to digitize the evolution of Hamiltonian 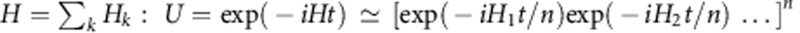, with each part implemented using single- and two-qubit gates (*ℏ*=1). We benchmark the simulation by comparing the experimental results with the exact digital outcome. Discretization unavoidably leads to deviations, and the digital errors are quantified in Supplementary Note 4.

**Figure 4:**
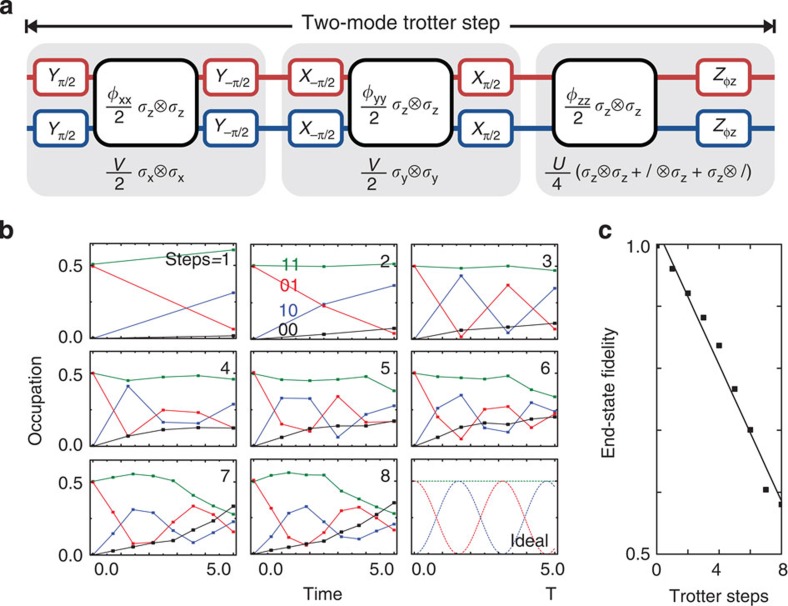
Simulation of two fermionic modes. (**a**) Construction of the two-mode Trotter step, showing the separate terms of the Hamiltonian (equation (2)). See Supplementary Note 1 for the pulse sequence and gate count. (**b**) Occupation of the modes versus simulated time for *n*=1,...,8 steps. Colour coding denotes the state probabilities. Input state is 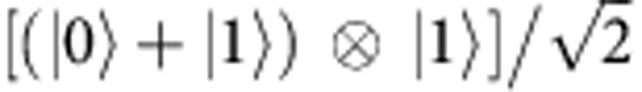, and *V*=*U*=1. The ideal dependence is shown in the bottom right. The final simulation time is *T*=5. (**c**) The end-state fidelity decreases with step by 0.054, following a linear trend.

We start by visualizing the kinetic interactions between two fermionic modes. The construction of the Trotter step is shown in Fig. 4a and directly follows from the Hamiltonian in equation (2). The step consists of the 
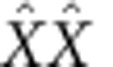
, 
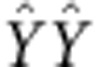
 and 
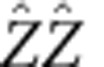
 terms, constructed from 
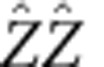
 terms and single-qubit rotations. We simulate the evolution during time Δ*t* by setting *φ*_*xx*_=*φ*_*yy*_=*V*Δ*t* and *φ*_*z*_=*φ*_*zz*_=*U*Δ*t*/2, and using *V*=*U*=1. We evolve the system to a time of *T*=5.0, and increase the number of steps (Δ*t*=*T*/*n*, with *n*=1,...,8). The data show hallmark oscillations, Fig. 4b, indicating that the modes interact and exchange excitations. We find that the end-state fidelity, taken at the same simulated time, decreases approximately linearly by 0.054 per step (Fig. 4c).

The above example shows that fermionic simulations, clearly capturing the dynamics arising from interactions, can be performed digitally using single-qubit gates and the tunable CZ_*φ*_ gate. Moreover, increasing the number of steps improves the time resolution, but at the price of increasing errors. A crucial result is that the per-step decrease in the end-state fidelity is consistent with the gate fidelities. Using the typical values of 7.4 × 10^−3^ entangling gate error and 8 × 10^−4^ single-qubit gate error as previously determined for this platform^12^, we arrive at an expected Trotter step process error of 0.07, considering the step consists of six entangling gates and 28 single-qubit gates (including X, Y rotations as well as idles). In addition, we have determined the Trotter step gate error in a separate interleaved randomized benchmarking experiment (Supplementary Note 3), and found a process error of 0.074, which is consistent with the observed per-step state error. We find that the process fidelity is thus a useful estimate, even though the simulation fidelity depends on the state and implemented model.

### Simulations with three and four fermionic modes

**Figure 5:**
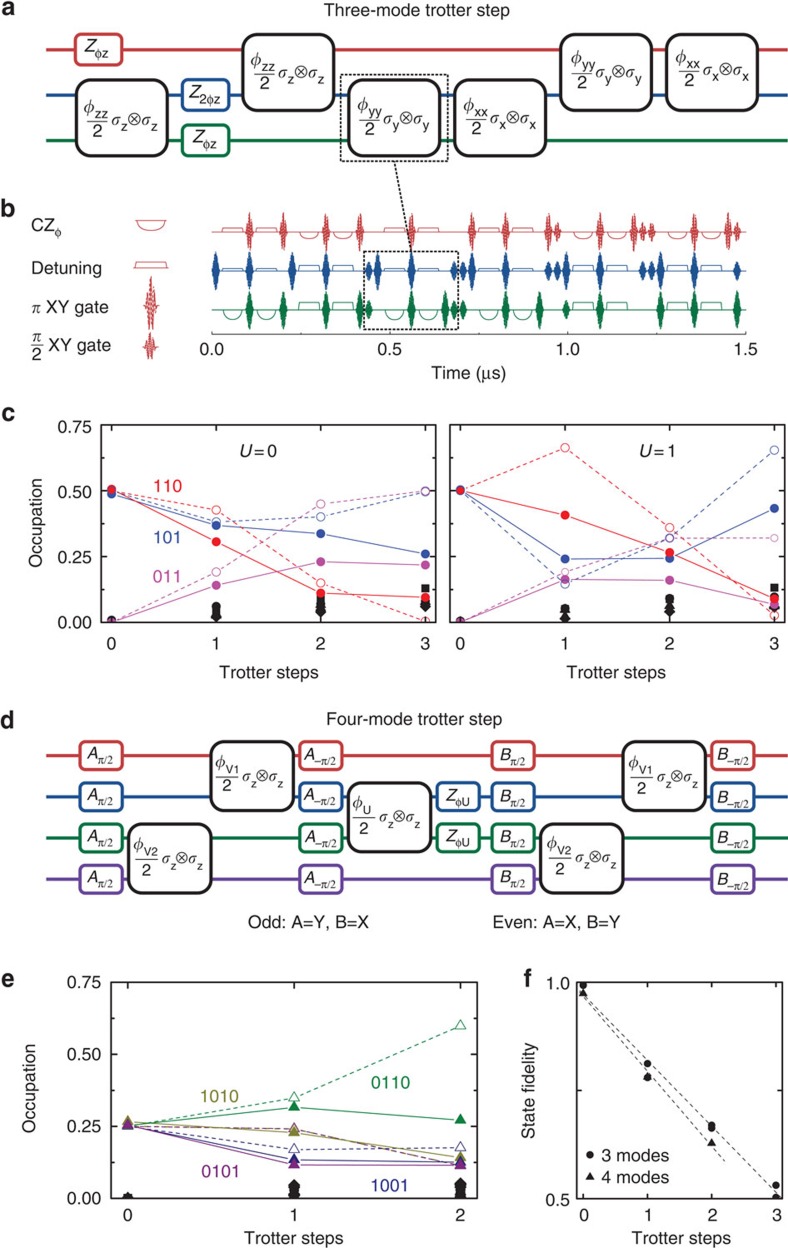
Fermionic models with three and four modes. (**a**) Three-mode Trotter step, with the Trotter step pulse sequence in **b**. The Trotter step consists of 12 entangling gates and 87 single-qubit gates (see text). The 
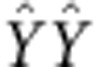
 interaction is highlighted (dashed). The amplitudes of the rotations are controlled by the values of *V* and *U*: 
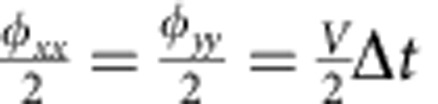
, and 
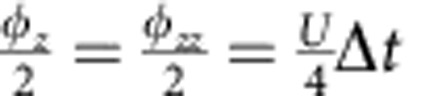
. (**c**) Simulation results for three modes with and without on-site interaction. Full symbols: experiment. Open symbols: ideal digitized. Black symbols: population of other states. Input state is 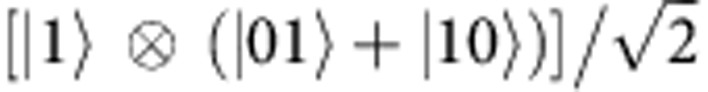, and *V*=1. (**d**) Construction of the four-mode Trotter step. The amplitudes of the rotations are: 
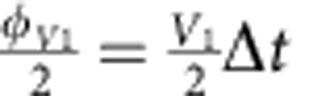
, 
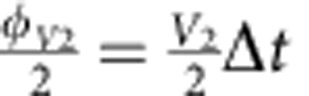
 and 
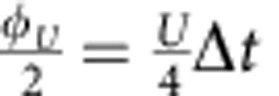
. (**e**) Four-mode simulation results for *V*_1_=*V*_2_=1, *U*_23_=1 and *U*_14_=0. Input state is 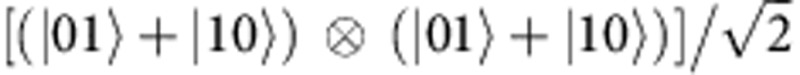. (**f**) Fidelities versus Trotter step for the three-mode simulation (dots) and the four-mode simulation (triangles).

Simulations of fermionic models with three and four modes are shown in Fig. 5. The three-mode Trotter step and its pulse sequence are shown in Fig. 5a,b. An implementation of the 
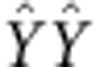
 gate is highlighted: the top qubit (red) is passive and detuned away, the middle qubit (blue) is tuned to an optimal frequency for the interaction, and the bottom qubit (green) performs the adiabatic trajectory. *π*-pulses on the passive qubit suppress dephasing and parasitic interactions. Figure 5c shows the simulation results for *V*=1, *U*=0 (hopping only) and *V*=1, *U*=1 (with on-site repulsion). Input state generation is shown in Supplementary Note 1. The simulation data (closed symbols) follows the exact digital outcome (open symbols), accumulating a per-step error of 0.15 (Fig. 5f) and gradually populating other states (black symbols). The fidelity is the relevant figure of merit; the per-step error being the same for different model parameters indicates that the simulation outcomes are distinct.

For the four-mode experiment, we simulate an asymmetric variation on the Hubbard model. Here the repulsive interaction is between the middle modes only (right well in Fig. 1a), while the hopping terms are kept equal. Asymmetric models are used in describing anisotropic fermionic systems^26^. In addition, the simulation can be optimized: gate count is reduced by the removal of interaction between the top and bottom modes, and the Trotter expansion can be rewritten in terms of odd and even steps such that the starting and ending single-qubit gates cancel (Supplementary Note 6). The Trotter step is shown in Fig. 5d. The results are plotted in Fig. 5e. We find that the state fidelity decreases by 0.17 for the four-mode simulation, see Fig. 5f.

The three- and four-mode experiments underline that fermionic models can be simulated digitally with large numbers of gates. The three-mode simulation uses in excess of 300 gates. We perform three Trotter steps, and per step we use: 12 entangling gates, 53 microwave *π* and *π*/2 gates, 19 idle gates, 3 single-qubit phase gates and for the non-participating qubit during the entangling operation: 12 frequency-detuning gates where phases need to be accurately tracked. Using the above typical errors for gates, we arrive at an estimated process error of 0.16 for the three-mode simulation, and an error of 0.15 for the four-mode simulation (per four-mode Trotter step: 10 entangling gates and 98 single-qubit gates). The process errors are close to the observed drop in state fidelity. The data are summarized in Table 1. Importantly, these results strongly suggest that the simulation errors scale with the number of gates, not qubits (modes), which is a crucial aspect of scalably implementing models on our platform. Therefore, the appreciable drop in total fidelity is currently the optimal for any quantum platform considering the large number of gates that we have implemented in this experiment. Moreover, the precision achieved in our experiment allows us to observe the expected fermionic behaviour at every Trotter step of the implemented protocol.

### Time-varying interactions

**Figure 6:**
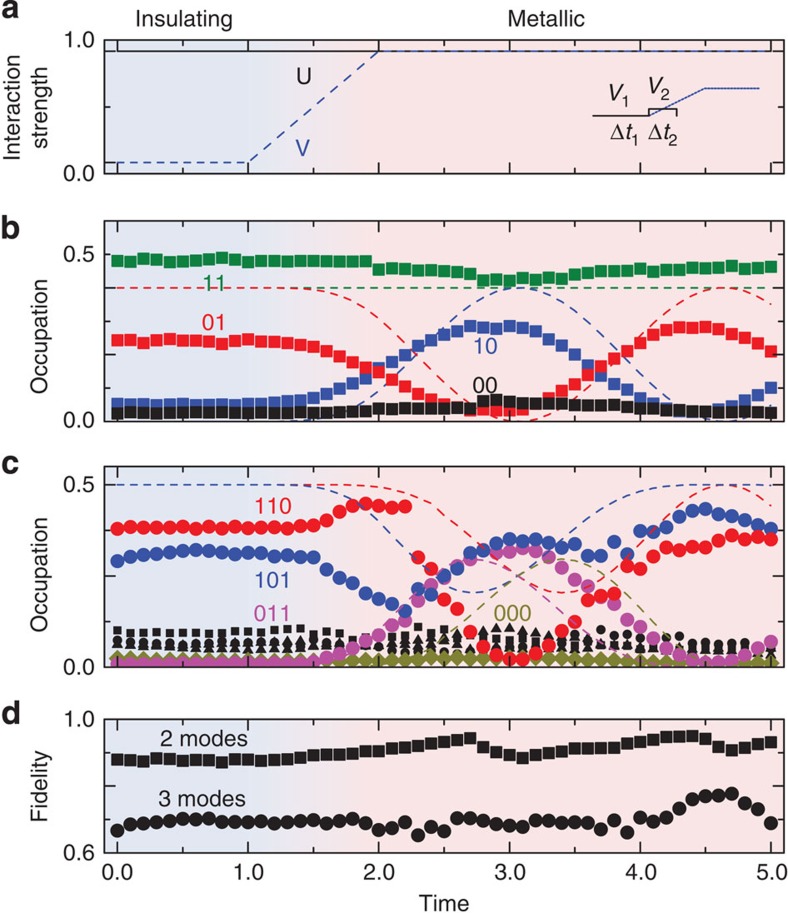
Simulations with time-varying interactions. (**a**) The system is changed from an insulating state (denoted by the blue background) to a conducting phase (denoted by a red background), by ramping the hopping term *V* from zero to one. Solid line: *U*, dashed line: *V*. Inset shows the choice of digitization on the ramp for the two-mode simulation. (**b**) Two-mode simulation showing dynamic behaviour starting at the onset of the *V* ramp. Dashed lines denote the ideal digitized evolution. (**c**) Three-mode simulation, showing non-trivial dynamics when the hopping term is non-zero. Dashed lines denote the ideal digitized evolution. Black symbols indicate the population of other states. (**d**) Simulation fidelities.

We now address the simulation of fermionic systems with time-dependent interactions. In Fig. 6a, we show an experiment where we ramp the hopping term *V* from 0 to 1 while keeping the on-site repulsion *U* at 1, essentially changing the system from an insulating to a metallic phase. This transition is simulated for two modes using two Trotter steps, see inset, and with one step for three modes. For the latter case, we take the average of *V* over the relevant time domain. The data are shown in Fig. 6b,c, and clearly mirror the dynamics of the hopping term. At time smaller than 1.0, the system is frozen and the mode occupations are virtually unchanged, reflecting the insulating state. Interactions become visible when hopping is turned on, effectively melting the system, and follow the generic features of the exact digital outcome (dashed). The simulation fidelities lie around 0.9–0.95 for two modes and 0.7–0.8 for three modes, see Fig. 6d. These fidelities are around or somewhat below those for time evolution with constant interactions, presumably due to control errors related to parasitic qubit interactions, which also lead to the populating of other states (black symbols). The dynamic simulation highlights the possibilities of exploring parameter spaces and transitions with few steps.

## Discussion

We have demonstrated the digital quantum simulation of fermionic models. Simulation fidelities are close to the expected values, and with improvements in gates and architecture, the construction of larger testbeds for fermionic systems appears viable. Moreover, a future implementation of quantum error correction in combination with these techniques will enable the efficient and scalable digital quantum simulation of fermionic models. Bosonic modes can be elegantly introduced by adding linear resonators to the circuit, establishing a fermion-boson analogue-digital system^15^,^16^ as a distinct paradigm for quantum simulation.

## Methods

### Experimental details

Experiments are performed in a wet dilution refrigerator with a base temperature of 20 mK. Qubit frequencies are chosen in a staggered pattern to minimize unwanted interaction. Typical qubit frequencies are 5.5 and 4.8 GHz. Exact frequencies are optimized based on the qubits' |*e*〉 and |*f*〉 state spectra along the fully tunable trajectory of the CZ_*φ*_-gate, as well as on minimizing the interactions between next-nearest neighbouring qubits. Used qubits are Q1–Q4 in ref. 19. Data are corrected for measurement fidelity, typical measurement errors are 0.01 for qubits Q1 and Q3 and 0.04 for Q2 and Q4 (refs 19, 27).

### State fidelity

The state fidelity is computed using 
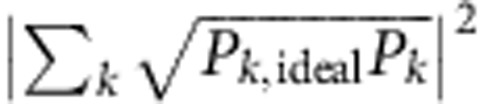
, which is equal to |〈Ψ_ideal_|Ψ〉|^2^ to first order. Here *P*_*k*,ideal_ and *P*_*k*_ are mode occupations and *k* runs over the computational basis. The consistency with measured process fidelities, and the scaling of the simulation fidelity with steps justify this approach.

## Additional information

**How to cite this article:** Barends, R. *et al*. Digital quantum simulation of fermionic models with a superconducting circuit. *Nat. Commun.* 6:7654 doi: 10.1038/ncomms8654 (2015).

## Supplementary Material

Supplementary Information Supplementary Figures 1-6, Supplementary Notes 1-6 and Supplementary References

## Figures and Tables

**Table 1:** Gate counts for the two-, three- and four-mode Trotter steps.

	Two-mode	Three-mode	Four-mode
Entangling CZ_*φ*_ gates	6	12	10
Single-qubit gates	28	87	98
Microwave *π* and *π*/2	20	53	56
Idle	6	19	22
Detuning	0	12	18
Virtual phase	2	3	2
est. Trotter step error	0.067	0.16	0.15
exp. Trotter step error	0.054	0.15	0.17

Est., estimated; exp., experimental.

We count idles as having the same duration as the microwave *π* and *π*/2 gates; this is the relevant approach for estimating total process fidelities. The gate counts are for a single Trotter step only, and exclude input state preparation. Estimated and experimental errors per Trotter step are tabulated at the bottom.
