# Supplementary material for: Digital quantum simulation of fermionic models with a superconducting circuit
Source: Nat Commun. 2015 Jul 8;6:7654. doi: 10.1038/ncomms8654 (PMC4510643; doi:10.1038/ncomms8654)
Supplement: Supplementary Information — Supplementary Figures 1-6, Supplementary Notes 1-6 and Supplementary References [file ncomms8654-s1.pdf]

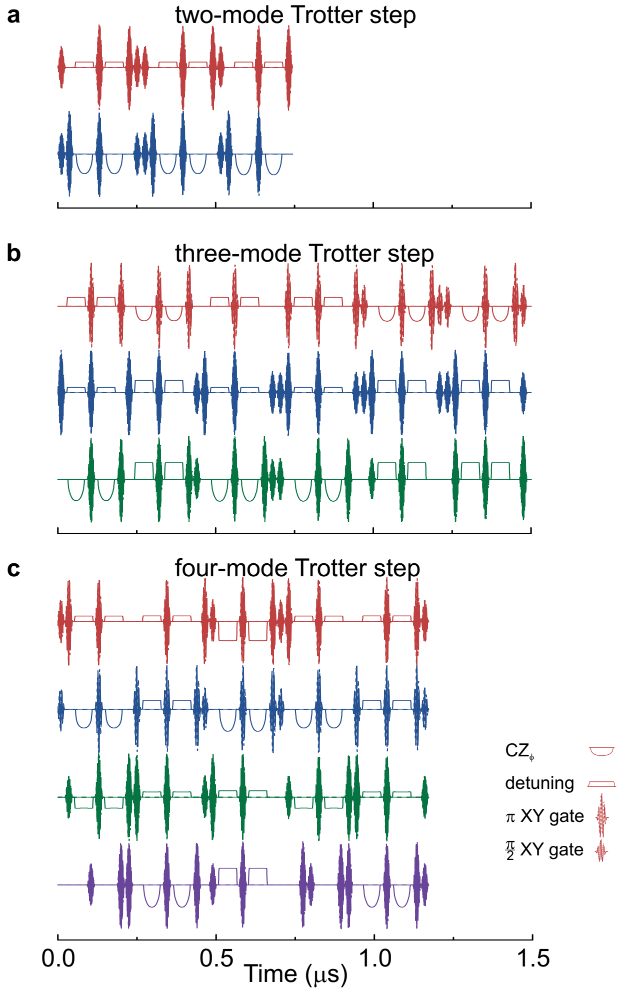

Supplementary Fig. 1. **Pulse sequences.** (a) Two-mode Trotter step. (b) Three-mode Trotter step. (c) Four-mode Trotter step. Shown are entangling gates as well as single-qubit microwave, idle and detuning gates. The legend is in the bottom right. XY pulses are 25 ns long.

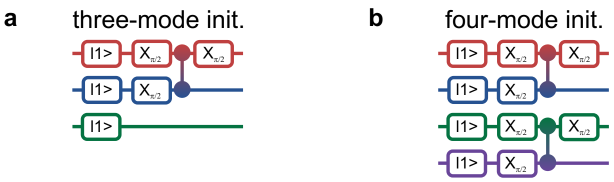

Supplementary Fig. 2. **Initialization gate sequence.** (a) Three-mode initialization. (b) Four-mode initialization.

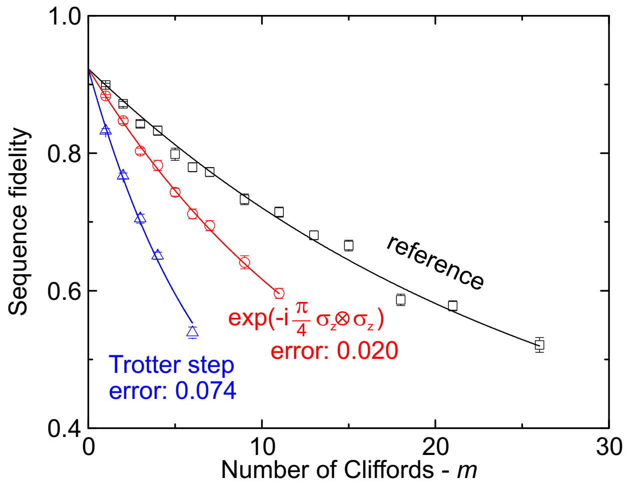

Supplementary Fig. 3. **Clifford-based randomized benchmarking of $\exp(-i\frac{\pi}{4}\sigma_z\otimes\sigma_z)$ and the two-mode Trotter step.** Sequence fidelity versus number of Cliffords. Black: reference. Colour: interleaved.

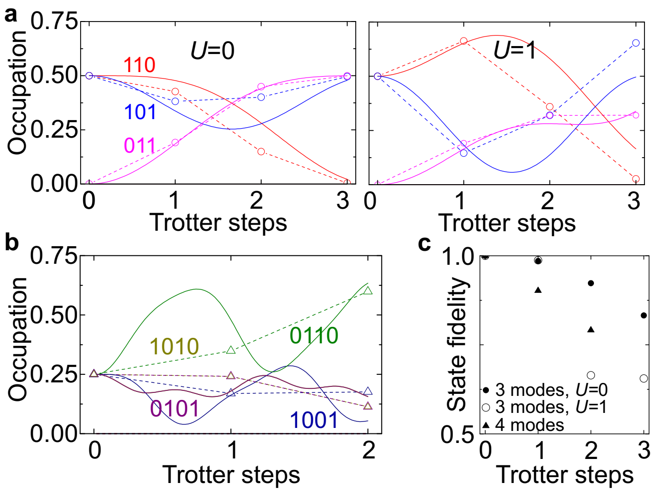

Supplementary Fig. 4. **Digital error for the time-independent simulation.** (a) Three mode simulation ($U = 0$, $U = 1$, $V = 1$). (b) Four mode simulation ($U_{23} = 1$, $U_{14} = 0$, $V = 1$). (c) Fidelity. Ideal evolution (solid lines) and exact digital solution (open symbols connected by dashed lines).

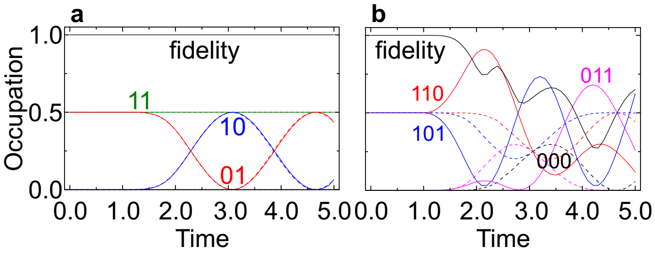

Supplementary Fig. 5. **Digital error for the time-dependent simulation.** (a) With two modes, and two Trotter steps. (b) With three modes, using one Trotter step. Ideal evolution (solid lines), exact digital solution (dashed lines), and fidelity (solid black).

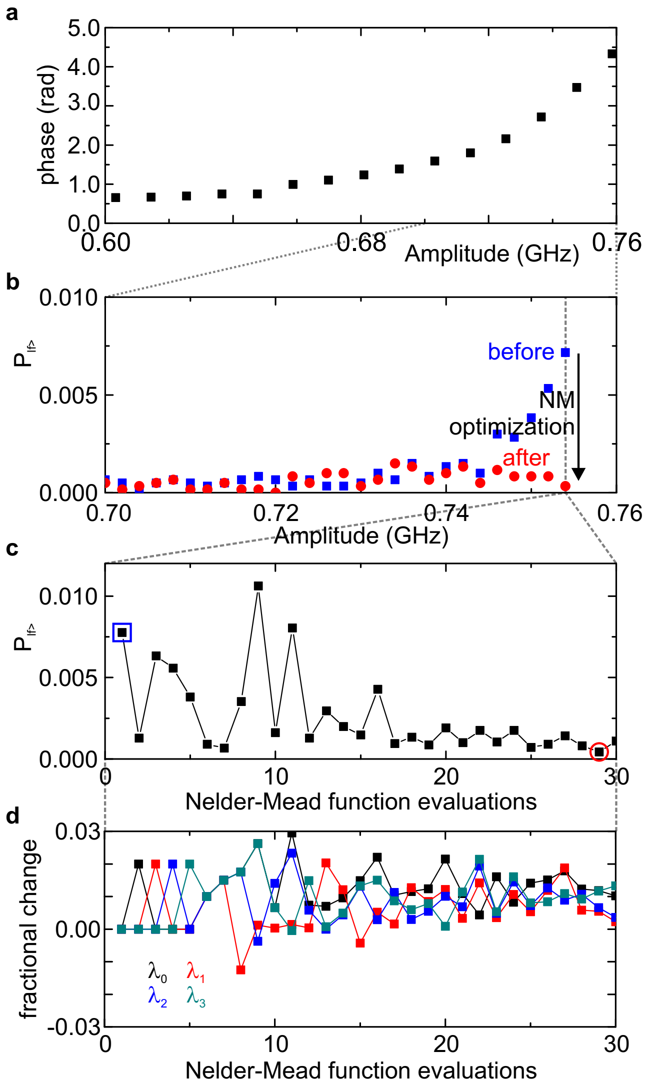

Supplementary Fig. 6. **Minimizing leakage of the CZ$_\phi$ gate.** (a) Tunable phase versus pulse amplitude, determined with quantum state tomography. (b) Zoom-in of the amplitude region for large phases, showing the $|f\rangle$-state population before (blue) and after (red) Nelder-Mead optimization. (c) Population of $|f\rangle$ versus Nelder-Mead function evaluation, showing a downwards trend. (d) Optimization of the waveform parameters with Nelder-Mead function evaluation, see Ref. [6] for the definition of these parameters.

Supplementary Note 1. TROTTER STEP PULSE SEQUENCES

A. Pulse sequences and gate counts

The two-, three- and four-mode Trotter step pulse sequences are shown in Supplementary Fig. 1.

B. Initialization

The gate sequences for the initialization of the three- and four-mode simulation are shown in Supplementary Fig. 2. For the two-mode simulation the input state is: $[(|0\rangle + |1\rangle) \otimes |1\rangle]/\sqrt{2}$, for three modes: $[|1\rangle \otimes (|01\rangle + |10\rangle)]/\sqrt{2}$, and for four modes: $[(|01\rangle + |10\rangle) \otimes (|01\rangle + |10\rangle)]/2$.

Supplementary Note 2. QUANTUM PROCESS TOMOGRAPHY

We use quantum process tomography to determine the χ matrix. We start by initializing the qubits into the ground state, and prepare input states by applying gates from {I, X/2, Y/2, X}$^{\otimes 2}$. The process output is reconstructed by applying gates from the same group, essentially obtaining the 16 output density matrices. The χ matrix is then determined using quadratic maximum likelihood estimation, using the MATLAB packages SeDuMi and YALMIP, while constraining it to be Hermitian, trace-preserving, and positive semidefinite; the estimation is overconstrained. Non-idealities in measurement and state preparation are suppressed by performing tomography on a zero-time idle.

The χ matrices for processes $U_1 = \exp(-i\frac{\pi}{2}(b_1 b_2^\dagger + b_2 b_1^\dagger))$ and $U_2 = \exp(-i\frac{\pi}{2}(b_1^\dagger b_2 + b_2^\dagger b_1))$ are determined experimentally, and the matrix of process $U_2 U_1$ is computed from the experimentally obtained matrices following Ref. [1].

The used quantum circuits are

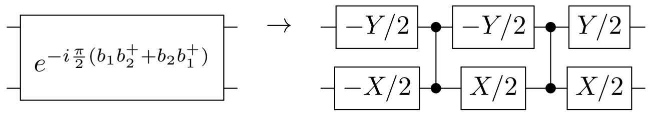

and

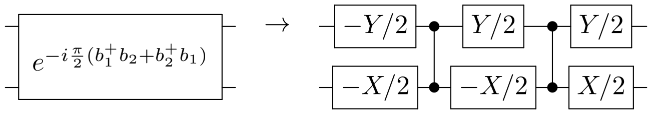

Supplementary Note 3. RANDOMIZED BENCHMARKING OF $\hat{Z}\hat{Z}$ AND THE TWO-MODE TROTTER STEP

The process fidelity of the $\exp(-i\frac{\phi}{2}\sigma_z \otimes \sigma_z)$ gate and the two-mode Trotter step are determined using interleaved Clifford-based randomized benchmarking [2–4]. This technique is insensitive to measurement and state preparation error, and determines the fidelity properly averaged over all input states, but it restricts the gates to have a unitary which lies within the group of Cliffords. As representative angles we have therefore used $\phi = \pi/2$, and $\phi_{xx} = \phi_{yy} = \phi_{zz} = \pi/2$ for the Trotter step.

The data are shown in Supplementary Fig. 3. We start by measuring the decay in sequence fidelity of sequences of random, two-qubit Cliffords (black symbols). When interleaving we see an extra decrease of sequence fidelity, which can be linked to the process fidelity of the interleaved gate. We find that the $\exp(-i\frac{\pi}{4}\sigma_z \otimes \sigma_z)$ gate and the Trotter step have errors of 0.020 and 0.074, respectively. We note that these values are consistent with estimation by adding individual gate errors (main Letter).

Supplementary Note 4. DIGITAL ERROR

The Trotter expansion introduces digital errors due to discretization. A full analysis of the digital error for the used model can be found in Ref. [5]. For the time-independent model, the two-mode simulation has zero digital error. For the three- and four-mode simulation the full evolution (solid lines), exact digital solution (open symbols connected by dashed lines), and fidelities due to digital error are shown in Supplementary Fig. 4.

For the time-dependent model we find a negligible digital error for two modes, and a significant error for three, see Supplementary Fig. 5. The large error for three modes arises from having to approximate a larger Hamiltonian, as well as using only a single step.

Supplementary Note 5. MINIMIZING LEAKAGE OF THE CZ$_\phi$ GATE

The tunable CZ$_\phi$ gate works by tuning the frequency of one of the qubits to approach the avoided level crossing of the $|ee\rangle$ and $|gf\rangle$ states, using an adiabatic trajectory [6]. For large phases we need to closely approach the avoided level crossing, inducing state leakage.

To minimize such leakage we have chosen to increase the length of the CZ$_\phi$ gate from a typical 40 ns [7] to 55 ns. However, for large phases (> 4.0 rads), see Supplementary Fig. 6a, we still see a considerable amount of leakage, see the Supplementary Fig. 6b. By choosing the leaked state population as a fitness metric, and using Nelder-Mead optimization in a similar approach to Ref. [8] to tune waveform parameters, see Supplementary Fig. 6c-d, we can significantly suppress leakage. We note that this optimization took approximately one minute in real time.

Supplementary Note 6. ASYMMETRIC HUBBARD MODEL

Here, we include the analysis of the fermionic asymmetric Hubbard model for 4 qubits employed in the Letter. Firstly, we present the model in terms of spin operators via the Jordan-Wigner transformation, and describe different limits of the

model. Secondly, we analyse the digital quantum simulation in terms of Trotter steps involving the optimized gates (CZ_ϕ).

The asymmetric Hubbard model (AHM) is a variation of the Hubbard model that describes anisotropic fermionic systems. Here, we are going to consider this model for two different fermionic species, that could represent spins, interacting with each other by the Coulomb term, and two lattice sites. The operators for this model have two indices, A_{ij}, where i and j indicate the site position and kind of particle, respectively. Since the fermions might have different masses, we have no reason to assume that the hopping terms will be the same. We can write the Hamiltonian for two sites, x and y, and two kinds of fermions, 1 and 2, as

$$\begin{aligned} H = &- V_1 \left(b^\dagger_{x1} b_{y1} + b^\dagger_{y1} b_{x1} \right) \\ &- V_2 \left(b^\dagger_{x2} b_{y2} + b^\dagger_{y2} b_{x2} \right) \\ &+ U_x b^\dagger_{x1} b_{x1} b^\dagger_{x2} b_{x2} \\ &+ U_y b^\dagger_{y1} b_{y1} b^\dagger_{y2} b_{y2}, \end{aligned} \tag{1}$$

where $b^\dagger_{mi}$ and b_{mi} are fermionic creation and annihilation operators of the kind of particle i for the site m. For the main Letter we use $b^\dagger_1$,$b^\dagger_2$,$b^\dagger_3$,$b^\dagger_4$, for $b^\dagger_{x1}$,$b^\dagger_{y1}$,$b^\dagger_{y2}$,$b^\dagger_{x2}$.

The Jordan-Wigner transformation will be used in our derivation to relate the fermionic and antifermionic operators with tensor products of Pauli matrices, which are operators that we can simulate in the superconducting circuit setup.

This transformation is based on a mapping between fermionic operators and spin-$1/2$ operators. In this case, the relations are

$$\begin{aligned} b^\dagger_{x1} &= \mathbb{I} \otimes \mathbb{I} \otimes \mathbb{I} \otimes \sigma^+ \\ b^\dagger_{y1} &= \mathbb{I} \otimes \mathbb{I} \otimes \sigma^+ \otimes \sigma^z \\ b^\dagger_{y2} &= \mathbb{I} \otimes \sigma^+ \otimes \sigma^z \otimes \sigma^z \\ b^\dagger_{x2} &= \sigma^+ \otimes \sigma^z \otimes \sigma^z \otimes \sigma^z. \end{aligned} \tag{2}$$

After this mapping, Hamiltonian (1) is rewritten in terms of spin-$1/2$ operators as

$$\begin{aligned} H = \; & \frac{V_1}{2} \left(\mathbb{I} \otimes \mathbb{I} \otimes \sigma^x \otimes \sigma^x + \mathbb{I} \otimes \mathbb{I} \otimes \sigma^y \otimes \sigma^y \right) + \frac{V_2}{2} \left(\sigma^x \otimes \sigma^x \otimes \mathbb{I} \otimes \mathbb{I} + \sigma^y \otimes \sigma^y \otimes \mathbb{I} \otimes \mathbb{I} \right) \\ &+ \frac{U_x}{4} \left(\sigma^z \otimes \mathbb{I} \otimes \mathbb{I} \otimes \sigma^z + \mathbb{I} \otimes \mathbb{I} \otimes \mathbb{I} \otimes \sigma^z + \sigma^z \otimes \mathbb{I} \otimes \mathbb{I} \otimes \mathbb{I} \right) \\ &+ \frac{U_y}{4} \left(\mathbb{I} \otimes \sigma^z \otimes \sigma^z \otimes \mathbb{I} + \mathbb{I} \otimes \mathbb{I} \otimes \sigma^z \otimes \mathbb{I} + \mathbb{I} \otimes \sigma^z \otimes \mathbb{I} \otimes \mathbb{I} \right), \end{aligned} \tag{3}$$

where the different interactions can be simulated via digital techniques in terms of single qubit and CZ_ϕ gates.

A. Gate decomposition

We consider the digital quantum simulation of the dynamics of Hamiltonian (3). The Trotter expansion consists of dividing the time t into n time intervals of length t/n, and applying sequentially the evolution operator of each term of the Hamiltonian for each time interval. In this case the evolution operators are associated with the different summands of the Hamiltonian.

In order to describe the digital simulation in terms of Trotter steps involving the optimized gates (CZ_ϕ), we will first consider the Hamiltonian in terms of $\exp[-i(\phi/2)\sigma^z \otimes \sigma^z]$ interactions. We take into account the relations

$$\begin{aligned} \sigma^x \otimes \sigma^x &= R_y(\pi/2) \sigma^z \otimes \sigma^z R_y(-\pi/2) \\ \sigma^y \otimes \sigma^y &= R_x(-\pi/2) \sigma^z \otimes \sigma^z R_x(\pi/2), \end{aligned} \tag{4}$$

where $R_j(\theta) = \exp(-i\frac{\theta}{2}\sigma^j)$ is the rotation along the j coordinate of a qubit. In these expressions the rotations are applied on the two qubits of the product.

The evolution operator associated with Hamiltonian (3) in terms of $\exp[-i(\phi/2)\sigma^z \otimes \sigma^z]$ interactions is

$$\begin{aligned} e^{-iHt} &\approx \prod_k \left(e^{-iH_k \frac{t}{n}} \right)^n \\ &\approx \Bigg(R_y(\pi/2) e^{-i\frac{V_1}{2} \mathbb{I} \otimes \mathbb{I} \otimes \sigma^z \otimes \sigma^z \frac{t}{n}} R_y(-\pi/2) R_x(-\pi/2) e^{-i\frac{V_1}{2} \mathbb{I} \otimes \mathbb{I} \otimes \sigma^z \otimes \sigma^z \frac{t}{n}} R_x(\pi/2) \\ &\quad \cdot R_y(\pi/2) e^{-i\frac{V_2}{2} \sigma^z \otimes \sigma^z \otimes \mathbb{I} \otimes \mathbb{I} \frac{t}{n}} R_y(-\pi/2) R_x(-\pi/2) e^{-i\frac{V_2}{2} \sigma^z \otimes \sigma^z \otimes \mathbb{I} \otimes \mathbb{I} \frac{t}{n}} R_x(\pi/2) \\ &\quad \cdot e^{-i\frac{U_x}{4} \sigma^z \otimes \mathbb{I} \otimes \mathbb{I} \otimes \sigma^z \frac{t}{n}} e^{-i\frac{U_x}{4} \mathbb{I} \otimes \mathbb{I} \otimes \mathbb{I} \otimes \sigma^z \frac{t}{n}} e^{-i\frac{U_x}{4} \sigma^z \otimes \mathbb{I} \otimes \mathbb{I} \otimes \mathbb{I} \frac{t}{n}} \\ &\quad \cdot e^{-i\frac{U_y}{4} \mathbb{I} \otimes \sigma^z \otimes \sigma^z \otimes \mathbb{I} \frac{t}{n}} e^{-i\frac{U_y}{4} \mathbb{I} \otimes \mathbb{I} \otimes \sigma^z \otimes \mathbb{I} \frac{t}{n}} e^{-i\frac{U_y}{4} \mathbb{I} \otimes \sigma^z \otimes \mathbb{I} \otimes \mathbb{I} \frac{t}{n}} \Bigg)^n. \end{aligned} \tag{5}$$

Note that, in principle, the ordering of the gates inside a Trotter step does not have a sizable effect as far as there are enough Trotter steps. Here, the number of Trotter steps is limited (n approximately ≤ 10) and different orderings will have different results. The different values in the orderings differ in a $O(1)$ constant, while the global digital error depends on the number of Trotter steps n as $1/n$ (the difference in errors due to different orderings does not depend on n).

If we consider the Trotter error, the fidelity could increase with an optimal ordering where we group terms of the Hamiltonian that commute with each other. Nevertheless, from the experimental point of view, the operators can be rearranged in a more suitable way in order to optimize the number of gates and eliminate global phases. In this sense, we must look for the optimal ordering by considering both aspects.

Here, we simply rearrange the operators in order to optimize the number of gates. If we consider that $R_j(\alpha) + R_j(\beta) = R_j(\alpha+\beta)$, then

$$
\begin{aligned}
e^{-iHt} \approx \prod_{i=1}^{n/2} \Bigg(& R_y^{'}(\pi/2) e^{-i\frac{V_1}{2}\mathbb{I}\otimes\mathbb{I}\otimes\sigma^z\otimes\sigma^z\frac{t}{n}} R_y^{'}(-\pi/2) R_y(\pi/2) e^{-i\frac{V_2}{2}\sigma^z\otimes\sigma^z\otimes\mathbb{I}\otimes\mathbb{I}\frac{t}{n}} R_y(-\pi/2) \\
& \cdot\, e^{-i\frac{U_x}{4}\sigma^z\otimes\mathbb{I}\otimes\mathbb{I}\otimes\sigma^z\frac{t}{n}} e^{-i\frac{U_x}{4}\mathbb{I}\otimes\mathbb{I}\otimes\mathbb{I}\otimes\sigma^z\frac{t}{n}} e^{-i\frac{U_x}{4}\sigma^z\otimes\mathbb{I}\otimes\mathbb{I}\otimes\mathbb{I}\frac{t}{n}} \\
& \cdot\, e^{-i\frac{U_y}{4}\mathbb{I}\otimes\sigma^z\otimes\sigma^z\otimes\mathbb{I}\frac{t}{n}} e^{-i\frac{U_y}{4}\mathbb{I}\otimes\mathbb{I}\otimes\sigma^z\otimes\mathbb{I}\frac{t}{n}} e^{-i\frac{U_y}{4}\mathbb{I}\otimes\sigma^z\otimes\mathbb{I}\otimes\mathbb{I}\frac{t}{n}} \\
& \cdot R_x^{'}(-\pi/2) e^{-i\frac{V_1}{2}\mathbb{I}\otimes\mathbb{I}\otimes\sigma^z\otimes\sigma^z\frac{t}{n}} R_x^{'}(\pi/2) R_x(-\pi/2) e^{-i\frac{V_2}{2}\sigma^z\otimes\sigma^z\otimes\mathbb{I}\otimes\mathbb{I}\frac{t}{n}} R_x(\pi/2) \Bigg)_{2i-1} \\
& \cdot \Bigg(R_x^{'}(-\pi/2) e^{-i\frac{V_1}{2}\mathbb{I}\otimes\mathbb{I}\otimes\sigma^z\otimes\sigma^z\frac{t}{n}} R_x^{'}(\pi/2) R_x(-\pi/2) e^{-i\frac{V_2}{2}\sigma^z\otimes\sigma^z\otimes\mathbb{I}\otimes\mathbb{I}\frac{t}{n}} R_x(\pi/2) \\
& \cdot\, e^{-i\frac{U_x}{4}\sigma^z\otimes\mathbb{I}\otimes\mathbb{I}\otimes\sigma^z\frac{t}{n}} e^{-i\frac{U_x}{4}\mathbb{I}\otimes\mathbb{I}\otimes\mathbb{I}\otimes\sigma^z\frac{t}{n}} e^{-i\frac{U_x}{4}\sigma^z\otimes\mathbb{I}\otimes\mathbb{I}\otimes\mathbb{I}\frac{t}{n}} \\
& \cdot\, e^{-i\frac{U_y}{4}\mathbb{I}\otimes\sigma^z\otimes\sigma^z\otimes\mathbb{I}\frac{t}{n}} e^{-i\frac{U_y}{4}\mathbb{I}\otimes\mathbb{I}\otimes\sigma^z\otimes\mathbb{I}\frac{t}{n}} e^{-i\frac{U_y}{4}\mathbb{I}\otimes\sigma^z\otimes\mathbb{I}\otimes\mathbb{I}\frac{t}{n}} \\
& \cdot R_y^{'}(\pi/2) e^{-i\frac{V_1}{2}\mathbb{I}\otimes\mathbb{I}\otimes\sigma^z\otimes\sigma^z\frac{t}{n}} R_y^{'}(-\pi/2) R_y(\pi/2) e^{-i\frac{V_2}{2}\sigma^z\otimes\sigma^z\otimes\mathbb{I}\otimes\mathbb{I}\frac{t}{n}} R_y(-\pi/2) \Bigg)_{2i},
\end{aligned}
\tag{6}
$$

where we use the prime notation in the rotation to distinguish between gates applied on different qubits. This decomposition between even and odd Trotter steps is suitable in order to simplify rotations in x and y, and, therefore, avoid higher number of gates.

The sequence of gates for one odd Trotter step in the digital simulation of the Hubbard model with four qubits is

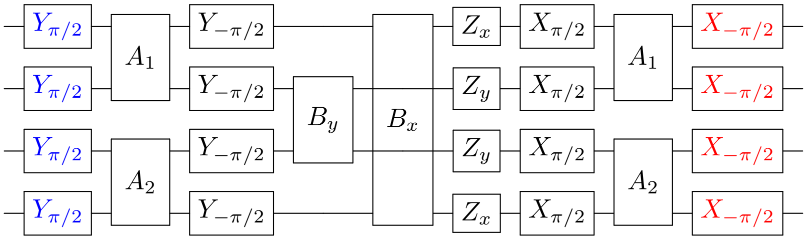

and for one even Trotter step:

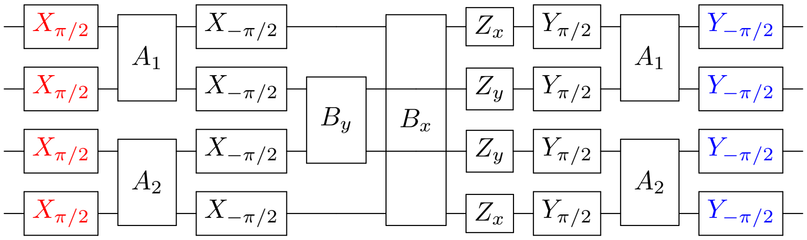

The gates A_i and B_j are two-qubit gates in terms of the $\exp[-i(\phi/2)\sigma^z \otimes \sigma^z]$ interactions: $A_i = \exp(i\frac{V_i}{2}\sigma^z \otimes \sigma^z \frac{t}{n})$ and $B_j = \exp(-i\frac{U_j}{4}\sigma^z \otimes \sigma^z \frac{t}{n})$. The Z_i gates are single qubit rotations: $Z_i = \exp(-i\frac{U_i}{4}\sigma^z \frac{t}{n})$, and X_α and Y_α are rotations along the x and y axis, respectively.

The $\exp[-i(\phi/2)\sigma^z \otimes \sigma^z]$ interaction can be implemented in small steps with optimized CZ_ϕ gates. The interaction is

$$e^{-i\frac{\phi}{2}\sigma^z \otimes \sigma^z} = \begin{pmatrix} 1 & & & \\ & e^{i\phi} & & \\ & & e^{i\phi} & \\ & & & 1 \end{pmatrix}.$$

The quantum circuits for simulating this are shown in the main Letter.

B. Particular case of the model

In order to avoid the gate B_x between the first and the fourth qubit, we can consider a particular case of the asymmetric Hubbard model, where $U_x = 0$. In this case, the circuit is the same but without the B_x and the Z_x gates. That is, for one odd Trotter step:

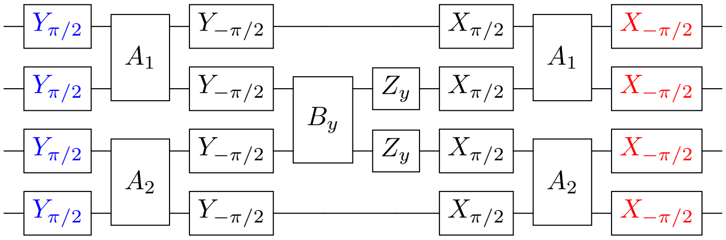

and for one even Trotter step:

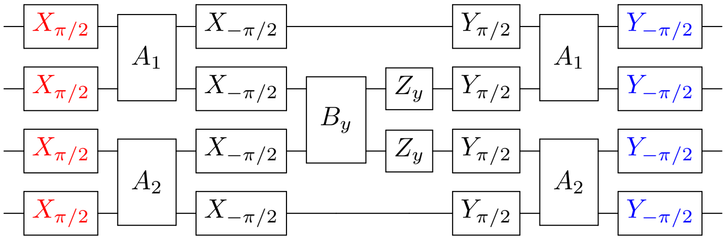

It is important to note that, for $n = 2$ Trotter steps, the red gates cancel each other, and we reduce the number of gates that should be applied. For $n > 2$, the blue gates also cancel each other except in the beginning and in the end of the quantum simulation.

C. Digital quantum simulation of the model

The relation among the values of the parameters in the numerical simulations and the values of the phases in the gates is the following

$$A_1 = \exp(-i\tfrac{V_1}{2}\sigma^z \otimes \sigma^z \tfrac{t}{n}) \;\rightarrow\; \Phi_{A_1} = \tfrac{V_1}{2}\tfrac{t}{n}$$

$$A_2 = \exp(-i\tfrac{V_2}{2}\sigma^z \otimes \sigma^z \tfrac{t}{n}) \;\rightarrow\; \Phi_{A_2} = \tfrac{V_2}{2}\tfrac{t}{n}$$

$$B_y = \exp(-i\tfrac{U_y}{4}\sigma^z \otimes \sigma^z \tfrac{t}{n}) \;\rightarrow\; \Phi_{B_y} = \tfrac{U_y}{4}\tfrac{t}{n}$$

$$Z_y = \exp(-i\tfrac{U_y}{4}\sigma^z \tfrac{t}{n}) \;\rightarrow\; \Phi_2 = \tfrac{U_y}{4}\tfrac{t}{n}\,.$$

Notice that we consider $\hbar = 1$ in the numerical simulations.

In summary, the fermionic asymmetric Hubbard model with two excitations, one for each kind of fermion, has been analysed and expressed in terms of simulatable spin operators. We have considered the digital quantum simulation in terms of Trotter steps involving the optimized gates (CZ_ϕ). This is the four-mode system experimentally simulated in the main Letter.

SUPPLEMENTARY REFERENCES

[1] Korotkov, A. N. Error matrices in quantum process tomography. Preprint at http://arxiv.org/abs/1309.6405 (2013).

[2] Ryan, C. A., Laforest, M., and Laflamme, R. Randomized benchmarking of single-and multi-qubit control in liquid-state NMR quantum information processing. *New J. Phys.* **11**, 013034 (2009)

[3] Brown, K. R. *et al.* Single-qubit-gate error below 10^{-4} in a trapped ion. *Phys. Rev. A.* **84**, 030303 (2011).

[4] Corcoles, A. D. *et al.* Process verification of two-qubit quantum gates by randomized benchmarking. *Phys. Rev. A* **87**, 030301(R) (2013).

[5] Las Heras, U., García-Álvarez, L., Mezzacapo A., Solano E., and Lamata, L. Fermionic Models with Superconducting Circuits. *EPJ Quantum Technology* **2**, 8 (2015).

[6] Martinis, J. M. and Geller, M. R. Fast adiabatic qubit gates using only σ_z control. *Phys. Rev. A* **90**, 022307 (2014).

[7] Barends, R. *et al.* Superconducting quantum circuits at the surface code threshold for fault tolerance. *Nature* **508**, 500-503 (2014).

[8] Kelly, J. *et al.* Optimal quantum control using randomized benchmarking, *Phys. Rev. Lett.* **112**, 240504 (2014).
